# Improvement of voice quality and prevention of deafness by a bone-conduction device

**DOI:** 10.1080/13102818.2014.949041

**Published:** 2014-10-22

**Authors:** Hyung-Woo Park, Myung-Sook Kim, Myung-Jin Bae

**Affiliations:** ^a^ Department of Transportation Environmental Research, Korea Railroad Research Institute, Uiwang-si, Gyeonggi-do, Korea; ^b^ Department of English Language and Literature, Soongsil University, DongJak-Ku, Seoul, Korea; ^c^ Electrical Engineering Department, Soongsil University, DongJak-Ku, Seoul, Korea

**Keywords:** bone-conduction, noise-induced hearing loss, speech quality enhancement, noise reduction

## Abstract

In modern society, people are involuntarily being exposed to various noises in their everyday-life environments. The increasing use of mobile phones and other portable devices as a primary means of communication outside of homes makes the current noise condition even worse. During the exchange of information on these devices, the volume is usually set 15 dB higher than the surrounding noise in order for the sound to be perceived more clearly. Hence, the sum of noise on these devices is usually estimated to be around 110 dB. This level of noise can cause noise-induced hearing impairment or even hearing loss to users when continued for a long time. A bone-conduction system can be a possible solution to reducing the noise while enhancing the quality of voice signals in mobile phones. In this study, we suggest that the implementation of the bone-conduction feedback system in mobile phones will raise the ratio of signal to noise with about 17 dB, enhancing the quality of voice signals.

## Introduction

With recent advancements in information and communication technology, personal electronic devices, including mobile phones, portable media players (PMPs), and MP3 players (MP3s) are being widely used. These devices are used in various places, secure and insecure, quiet and loud, secluded and crowded surroundings. Whatever the circumstances are, people do not want to be disturbed by the surrounding noise during their conversations. Consequently, using a mobile phone in public areas naturally leads to increasing the receiver volume and speaking loudly.[1–3]

In a crowded and noisy environment, the average noise exceeds 80 dBA. Those who use their phones in such places tend to set the volume of their phones much louder than the surrounding noise. Since phone manufacturers provide consumers with phones that can generate sound with capacities higher than 100 dB to meet consumers’ needs, it is possible to have higher volume if necessary.[1–5] However, people are concerned about the possibility of mobile phones used at loud volume settings leading to noise-induced hearing impairment or even hearing loss.[5] The actual outbreak of noise-induced hearing loss depends on the degree of sound volume and the duration of exposure. Roughly speaking, we may generalize that continuous exposure to high volume leads to hearing loss.[6–10]

In this paper, we propose a solution based on improving the sound quality of mobile phones with a bone-conduction system. The bone-conduction system uses a bio-engineered technology that can be operated for long periods of time, without causing any discomfort, while transferring sound directly to our inner ears.[11] However, there is one weakness of bone conduction, namely that as the vibration of voice sound transfers through the bones and skin of our bodies, higher frequencies can be lost.[12] Hence, the study introduces a method that utilizes both normal mobile phone speakers and bone-conducted speakers in order to eliminate noise and to acquire better sound quality without losing high-frequency sound. The study proves that the overall quality of mobile phones would be improved with the bone-conduction system if proper experiments are conducted.

In a previous research, we conducted a pilot experiment adding the bone-conduction system to a mobile phone, in order to reduce noise interference, and we succeeded in improving the sound quality by raising the volume by 10 dB.[6,13] In this study, we use not only reduced ambient noise, but also amplified incoming sound, compensated and synthesized into combinatorial sound. In this study, we will examine the characteristics of the transferring process between human bodies and the bone-conduction system, and report the result of the experiment in which the bone-conduction system reduces the external noise and improved overall sound quality of mobile phones. The result of the experiment exceeded the performance of the previous study and raised the volume by about 6 dB, totalling for 17 dB.

## Materials and methods

### Subjects

Twenty healthy volunteers (10 male and 10 female) [5] aged from 24 to 32 years took part in the study. Declaration of informed consent was obtained from all participants.

### Human auditory sense and pure tone bone conduction: a brief overview

The human ear converts sound waves to electrochemical impulses and can separate sound through three parts: outer ear, middle ear and inner ear. Sound can be registered by human ears in two ways: first, through air conduction (normal hearing) and second, through bone conduction. In bone-conduction, the bones of the skull transfer the vibration of sound directly to the cochlea, the spiral-shaped part of the inner ear.[11] We can say that bone conduction transfers sound directly to the inner ear through the skull in several stages including vibrating cochlea, passing through the auditory nerve, and delivering the perceived sound to the brain.[10,12]

### Ambient noise during mobile phone calls: a brief overview

For the sake of efficiency, data were collected from several different locations and situations and at different time of the day (Table 1). The data were analysed through repeated recordings by utilizing the average sound pressure. The ambient noise of 44.1 kHz, 16 bit mono recorded with a microphone and a laptop, was used for recordings and analysis. Table 1 shows the average ambient noise. Data are mean values from measurements made 10 times every 5 minutes.[1]

**Table 1.** Locations and average noise levels.

Location	Level (dBA)	Location	Level (dBA)
Subway platform	85.7	Shopping mall	78.3
Bus Inside	80.4	Parks	55.1
Subway	80.9	In office	61.2
Road side	77.9		

Phone call environments can be noisy or quiet, depending on the situation. Noisy environments such as subway stations, bus stations, malls and other public places produced results with an average ambient noise of 80 dB, whereas environments known to be quiet such as parks and offices had an average ambient noise of 58 dB. The average difference in the average noise level between noisy and quiet places was about 20 dB.[2]

**Figure 1.**
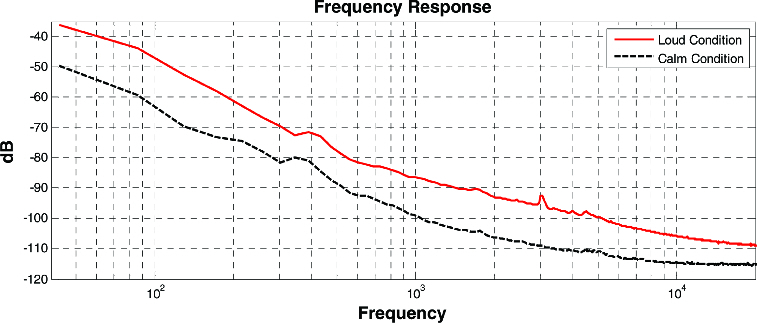
Ambient noise comparison between two places: loud condition (at public transportation, red line) and calm condition (at parks, dashed line).[1,2]

Figure 1 shows the frequency spectrum in the two types of places as classified in Table 1. The high energy range of ambient noise observed in the places for public transportation was between 100 Hz and 2 kHz. This distribution can also be found in the energy range of the human voice. Additionally, this bandwidth can irritate human ears. Meanwhile, ambient noise from quiet places like offices, as shown in the right panel of Figure 1, gives an overall low energy distribution over a few concentrated areas.

### Sound pressure from mobile phones

In order to examine the sound pressure outputs from mobile phones, three different mobile phones (LG Electronics’ 3G mobile phones, Samsung) and a landline phone (LG) were used for comparative analysis. Other devices used for the experiment include a microphone and an audio analyser (B&K 2250 Torso) and the broadcast of weather forecast used as a sound source.

**Figure 2.**
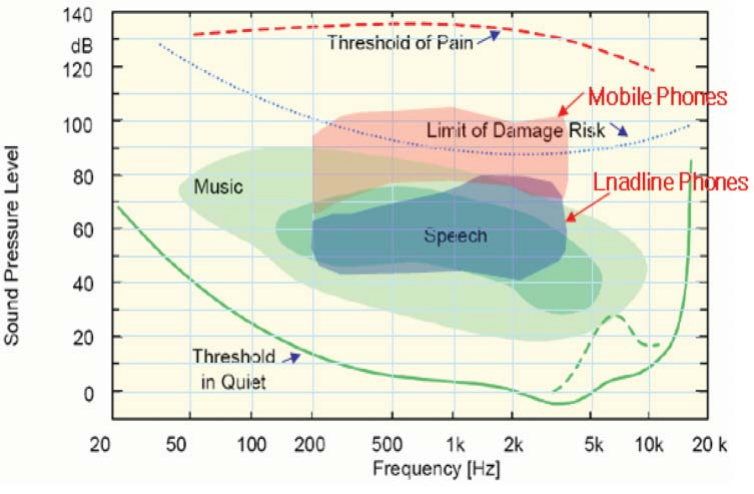
Auditory field.[7]

Comparative analysis was carried out with the frequencies obtained from the phones. Figure 2 shows the auditory field of human ears, as provided by the Occupational Safety and Health Research Institute (OSHRI) in 2007.[7] The auditory field is a graph illustrating the sound pressure distribution for each frequency near each person. According to OSHRI, the limit of damage risk for noise-induced hearing loss is estimated to be 88 dB in 2 kHz.[7] The analysed results show that mobile phones generate sound pressure of more than 100 dBA in 2 kHz while landline phones generated much less pressure, 80 dBA. Therefore, it could be expected that mobile phone users are at a greater risk of noise-induced hearing loss than landline phone users.[5–8,14–16]

The average difference between the maximum and minimum volume of a mobile phone was calculated to be 25 dB. The frequency outputs of both mobile and landline phones were measured between 200 Hz and 3.4 kHz. The maximum volume of mobile phones exceeded the limit of damage risk in the frequency area of 300 Hz to 3.4 kHz, covering most of the frequency ranges.

### Bone-conduction system

The bone-conduction system proposed in this study processes noise from an additional microphone and generates sound through an additional bone-conducting speaker in order to improve the sound quality. The speaker will vibrate the bones in the human skull to transfer sound waves for a better sound quality while using mobile phones. Regular mobile phones are built with a microphone and a speaker, but the bone-conduction system will use an additional microphone and speakers. Reducing the ambient noise by providing an average phone with a bone-conduction system will enhance its sound quality as well as conversation quality for the mobile phone users.

**Figure 3.**
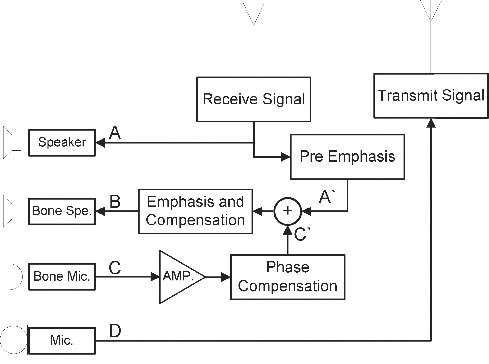
Block diagram of the bone-conduction system. A: voice signal in an ordinary mobile phone; B: modified signal from bone-conducting speakers (B = A′ + C′ + D); A′: amplified high frequency from received signal; C′: compensated signal of ambient noise phase; C: incoming signal from additional microphone; D: incoming signal from ordinary mobile phone microphone.

Figure 3 is a block diagram which outlines the method for building the bone-conduction system as proposed in this paper. An additional microphone receives the ambient noise, which is transferred through the bone-conduction noise cancellation system. The bone-conduction speaker generates the vibration of anti-phase noise and eventually reduces the noise level. In this system, the ambient noise is cancelled at the cochlea by applying destructive interference with the anti-phase of the environment noise. Then, the quality of voice signals is enhanced by undergoing constructive interference with the in-phase of pre-magnified signals.

In Figure 3, the A′ signal amplifies the high-frequency reception into harmonics, and the C′ signal receives the ambient noise through the bone-conducting microphone and compensates the signal. The composite signal of A′ and C′ is set as a modelling parameter and by adjusting its values with emphasis and compensation, and then, signal B enters through the bone-conducting speakers. The overall sound quality of the final output is enhanced through signal B by directly and indirectly affecting signals of A and C at the cochlea. The ambient noise is countervailed by the anti-phase output signal from the bone-conducting speakers. Furthermore, the high frequency in ordinary speakers is amplified and thus the final output to mobile-phone users will be clearer and louder as the speakers eliminate ambient noise and enhance sound quality.

## Results and discussion

Mobile phones can be used in various environments, including loud places like shopping malls and relatively quiet places like offices, which is why mobile phones are manufactured to generate sound that is loud enough to be audible in various locations. However, such sound pressure may cause noise-induced hearing loss and therefore users must take caution.

### Evaluation of performance

**Figure 4.**
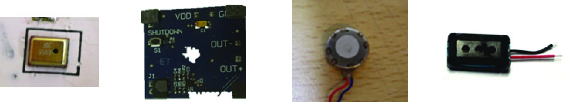
Components applied to the bone-conduction system.

In order to evaluate the performance of the mobile phone installed with a bone-conducting speaker as proposed in this paper, an experiment was conducted in an anechoic chamber where the background noise was set at 30 dB. Signals were measured by monitoring the values from an additional microphone and a vibration sensor attached to phones [17]. Parameters were based on the measurement of open and closed-loop frequency responses for modelling characteristics and bone-conduction compensation values. The sound quality of a mobile phone was confirmed by applying the frequency compensation model to the sound source. The system performance was evaluated by conducting a sensitivity assessment. The additional bone-conduction system was used to monitor noise by utilizing a microelectrical-mechanical system (MEMS) microphone for collecting sound and amplifying voltage with a preamplifier while reversing the phase to activate the bone-conducting speakers by using a power amplifier. Figure 4 pictures the components applied to the bone-conduction system.

### Installation of the bone-conduction system

**Figure 5.**
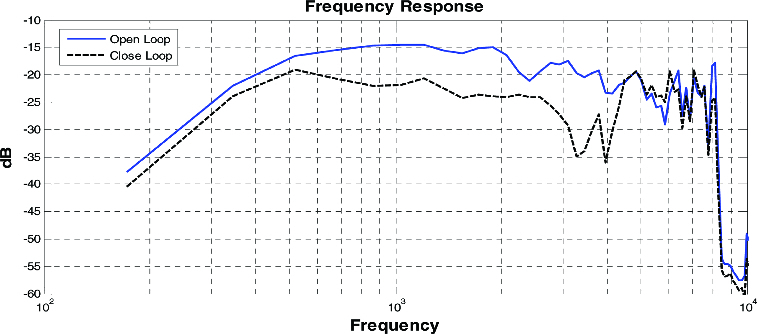
Open- and closed-loop frequency responses.

In order to install the bone-conduction system into an ordinary phone, the vibration motor originally built in the phone will be replaced with a similar sized bone-conducting speaker. To obtain modelling parameters, a sine wave was generated with an increase from 300 to 8000 Hz in the monitor speaker to measure the open- and closed-loop responses, while simultaneously measuring the input and output of the bone-conduction system. Figure 5 shows the measurement of frequency responses. The open-loop curve illustrates vibration values from the bone-conducting microphone to the speaker. On the other hand, the closed-loop curve indicates the effects on the bone-conduction feedback system from the human body. The closed-loop response shows a decrease of 5–10 dB to 600–4000 Hz. The high-frequency response of over 4000 Hz shows a disturbance in the resulting values due to a decrease of high frequency during the transfer of vibration to the bone and skin tissues.

**Figure 6.**
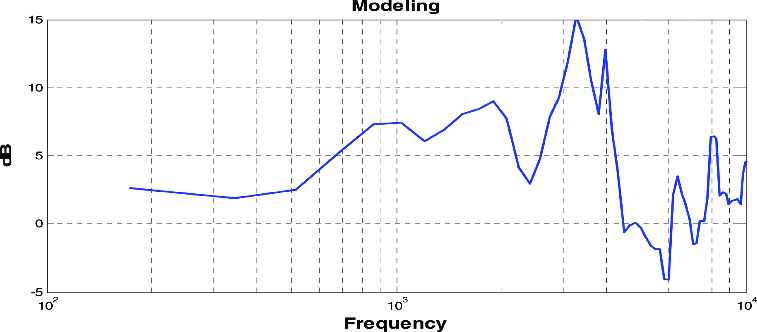
Parameters of frequency modelling.

Figure 6 illustrates the results from frequency modelling for compensating the bone-conduction feedback system, using the parameters obtained in Figure 5. The threshold power level of outputs from the bone-conduction system, which affects the human ear, was utilized and modified for enhancement of sound quality.

**Figure 7.**
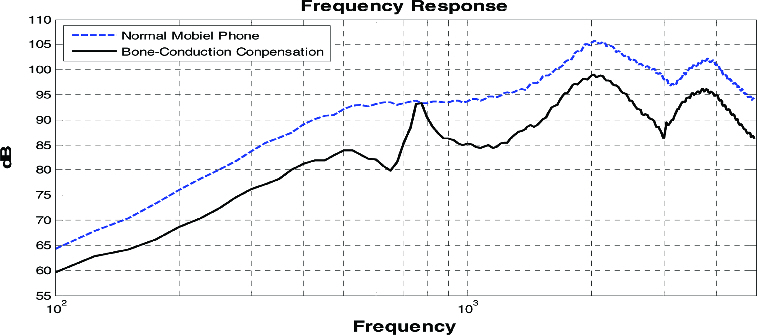
Comparison of frequency responses between an ordinary phone and a bone-conducting phone.

Figure 7 shows a comparison between the sound clarity of compensated sound signals from the bone-conduction system obtained with the modelling values in Figure 6, and the frequency responses from an ordinary mobile phone. Figure 7 actually illustrates the effect of compensation by a decrease in the damping ratio of high frequency in the output values of combining an ordinary and bone-conducting speaker over 1000 Hz. Particularly, compensation occurred in the frequency range between 300 and 5000 Hz.

**Figure 8.**
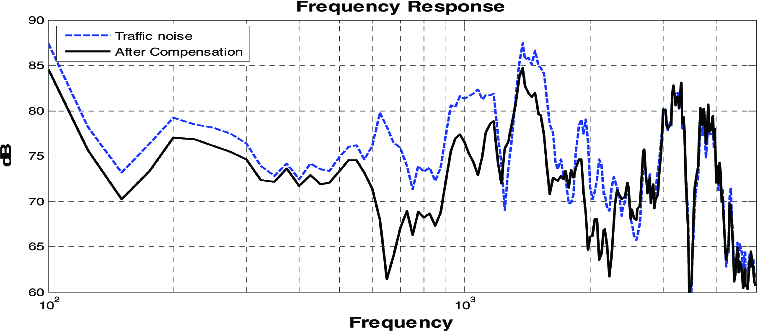
Comparative analysis before and after the activation of the proposed system.

Figure 8 shows the performance of the phones before and after the activation of the proposed system with respect to the actual amount of ambient noise. Frequency responses were measured by placing the mobile phone close to the ears of the participants using Torso. The environment was set with an average sound pressure of 80 dB. The proposed system achieved the maximum attenuation of 19 dB in noise interference at 700 Hz. Under 500 Hz, the high pass filter was used, eliminating ambient noise in a low-frequency range to about 3 dB.

### Sensitivity assessment

Sensitivity assessment was conducted in an anechoic room with the ambient noise condition of 80 dB. Clarity analysis was conducted using the bone-conduction feedback system while listening to the weather forecast as the incoming sound source of mobile phones. For sensitivity assessment, the results were obtained by using the value of the difference between the two appointed volumes as well as with the difference of volumes obtained with and without the use of bone-conduction system. Table 2 shows the results of emotional evaluation from 20 participants. In the ambient noise conditions at 80 dB, the average volume set by the participants was 99.95 dB. On the other hand, when the proposed system was activated, the average decreased to 83.55 dB, showing a 17 dB decrease but with clear communication. The maximum decrease in the volume was 22 dB and the minimum, 13 dB. The results showed an enhancement of 6 dB compared to the previous study, in which only the environment noise was neutralized by the bone-conducting speakers.[5] Further enhancement of 6 dB in volume was obtained by transferring combinatorial sound from magnifying and synthesizing the received signals using the bone-conducting feedback system.

**Table 2.** Results from the sensitivity assessment.

Evaluators	Before (dB)	After (dB)	Evaluators	Before (dB)	After (dB)
#1	99	85	#11	97	84
#2	102	84	#12	99	82
#3	98	74	#13	103	83
#4	97	82	#14	99	85
#5	99	83	#15	97	83
#6	103	89	#16	98	84
#7	101	83	#17	103	85
#8	99	81	#18	102	84
#9	98	83	#19	99	86
#10	101	84	#20	105	83
Average	99.95	83.35			

The results from our study are promising in the context of the ever increasing mobile-phone use and concerns about noise-induced hearing loss related to it. Furthermore, using phones can in itself add additional ambient noise to other people. Thus, the improvement of sound quality of mobile phones by reducing noise and enhancing incoming sound is necessary. Future efforts will be directed towards elimination of ambient noise through improving the response rate of the bone-conduction system.

## Conclusions

This study proposes a novel method for more effective noise reduction and magnification of speech signals of mobile phones by installing a bone-conduction system. The bone-conduction system can be implemented with ordinary phones only by installing additional bone-conducting speakers. The frequency responses from phones with the proposed system were analysed and a combinatorial system for transferring sound was modelled. Not only was the ambient noise neutralized with the anti-phase, but the incoming sound was amplified using in-phase signals, employing an improved method from previous studies. It was confirmed that through amplifying the incoming sound and using a combinatorial method, a further increase in sound volume can be obtained, as demonstrated by the 6 dB increase in this study. Future research in the proposed system will enhance the elimination of ambient noise through improving the response rate of the bone-conduction system.
